# A comparative evaluation of resin- and varnish-based surface protective agents on glass ionomer cement – a spectrophotometric analysis

**DOI:** 10.1080/26415275.2020.1711760

**Published:** 2020-01-11

**Authors:** Shreya Tyagi, Abi M. Thomas, Neeta Devi Sinnappah-Kang

**Affiliations:** a Department of Pedodontics and Preventive Dentistry, Christian Dental College, Ludhiana, India; b Betty Cowan Research and Innovation Centre, Christian Medical College and Hospital, Ludhiana, India

**Keywords:** Glass Ionomer cement, vaseline, GC Fuji VARNISH™, G-Coat Plus™, EQUIA^®^ Coat, Spectrophotometer

## Abstract

**Objectives:**

To evaluate and compare the effectiveness of resin- and varnish-based surface protective agents on Glass Ionomer Cement (GIC). The different surface protective agents used were: Vaseline^®^, GC Fuji VARNISH™ (varnish), G-Coat Plus™ (resin) and EQUIA^®^ Coat (resin).

**Method:**

Thirty-six identical specimens of GIC were made. Six specimens were used in preparation of standard solution and remaining thirty were divided into five groups with six specimens in each group. Each test specimen was coated with one of the surface protecting agent except for the control group. The specimens were immersed separately into 1 ml of 0.05% methylene blue solution for 24 h and then rinsed with deionised water and further immersed into tubes containing 1 ml of 65% nitric acid. Specimens, once completely dissolved in nitric acid solution, were filtered and centrifuged. The supernatant was used to determine the absorbance using a spectrophotometer. The effectiveness of the surface protecting agents for the GIC was recorded in micrograms of dye per specimen, where low values indicate good protection.

**Result:**

Tukey HSD test revealed that GC Fuji VARNISH™ (varnish; mean = 21.25 µg/ml), G-Coat Plus™ (resin; mean = 30.39 µg/ml) and EQUIA^®^ Coat (resin; mean = 9.32 µg/ml) were statistically not significantly different to each other and were effective in protecting the surface of GIC.

**Significance:**

The study found that there was a statistically significant difference between control and GC Fuji VARNISH™, G-Coat Plus™ and EQUIA^®^ Coat. The three agents were found to be equally effective in protecting the surface of GIC.

## 1. Introduction

With the advancements in adhesive restorative materials, modern dentistry is able to offer minimally invasive treatment procedures. Among the various adhesive restorative materials, Glass Ionomer Cement (GIC) is preferred by many clinicians due to its advantages of chemical adhesion to enamel and dentin, fluoride release and biocompatibility [1]. GIC holds an important position in restorative dentistry and thus, it is necessary to have a detailed understanding of its setting reaction and the methods to improve the physical properties of this material.

The setting reaction of GIC involves neutralization of the polyacid by the basic glass leading to the formation of metal polyacrylate units [2]. The setting reaction involves the following stages: decomposition of the powder, gelation, hardening and maturation [3]. When the acid attacks the surface of the glass it leads to decomposition of the powder and the release of metallic cations into the solution. A silica gel is then formed and this surrounds the unreacted glass particles. The ions that are released from the glass powder are Na^+^ and Ca^2+^ (or Sr^2+^), followed quickly by Al^3+^ ions. These cations react with the polymer chains of carboxylate groups and thus increase the viscosity and contribute to the gelation reaction. Hardening of the cement occurs as a result of formation of crosslinks between the polymer chains and metal cations. Initially there is formation of calcium polyacrylate within 45 s followed by aluminium polyacrylate formation in 10 min and this continues for approximately a day. After the initial hardening, further reactions continue for more than 24 h and this is known as maturation [3–5]. With maturation of the cement, the physical properties change, i.e. there is an increase in strength and translucency of the GIC.

Water plays an important role in the setting reaction. Initially it serves as the solvent for the polymeric acid and reaction medium for the setting reaction [6]. During the maturation stage, water becomes the component of the set GIC as tightly-bound water and its proportion increases with time for the first month [5].

The setting time of GIC is seven minutes from the start of the mix. The moisture isolation is crucial during this period [7]. According to Gemalmaz et al., the amount of soluble matrix is maximum during the early phases of GIC formation and the most sensitive period is the first six minutes after mixing. Any moisture contamination during this phase can cause the loss of soluble matrix and reduce its physical properties [8]. Hence the GIC should be protected from additional water contamination during the initial stages to prevent dissolution of ions whereas once it sets; it should be protected against dehydration to avoid cracking and crazing [9].

Much research has been done on the GIC surface protective agents. Earl et al. conducted a series of surface treatments in 1989 and showed that immediate covering of the immature cement surface with light activated bonding resin was the most effective method of limiting water movement across the surface [10]. Williams et al. in 1998 showed that there was no difference in the clinical efficacy of light cured resins and the conventional varnish in terms of strength and surface texture [11]. Gorseta et al. in 2016 reported that the flexural strength of GIC is improved by coating with varnish, followed by curing [12]. The present research work was done to compare the resin-based surface protective agents with varnish-based agent in protecting the surface of GIC. The different surface protective agents used for comparison with control (specimens with no protection) were Vaseline^®^, GC Fuji VARNISH™ (waterproof varnish), G-Coat Plus™ (resin) and EQUIA^®^ Coat (resin). The present study is unique in the sense that these four agents have not been compared in a single study.

## 2. Materials and methodology

In the present study, the capsule system of GIC (GC Fuji IX GP^®^ EXTRA) was used to prepare the specimens. Thirty-six identical specimens were prepared using stainless steel moulds with dimensions of 1.25 mm thickness and 8 mm internal diameter [Six of thirty-six specimens were used for the preparation of standard solutions and thirty specimens were divided into five groups with six specimens in each group to be coated with different agents]. The GC Fuji IX GP^®^ EXTRA (GC Corporation, Tokyo, Japan) capsules were activated and placed in an amalgamator for 10 s. GIC was dispensed directly into the stainless-steel moulds placed on a glass slab over a mylar strip (Samit^®^, New Delhi, India) with the help of a capsule applier (GC America Inc., Chicago, USA), taking care to avoid incorporation of air bubbles. The filled moulds were immediately covered with another mylar strip and a microscopic slide was laid over the top. This sandwich was held under the pressure of a glass slab to level the height of GIC with the mould and to produce a smooth surface. The specimens were allowed to remain between the glass slabs and the polyester strips for seven minutes to ensure complete curing of the cement. The excess material was removed with the help of a scalpel. After this step, the specimens were divided into different groups based on the surface protective agent used to coat the samples. The rationale of choosing the agents in this study was to compare the agents of three different classes: emollients (Vaseline^®^), solvent based water proof varnish (GC Fuji VARNISH™) and light cured resins (G-Coat Plus™, EQUIA^®^ Coat). Each group comprised of six specimens. Group I was uncoated and kept as control. Group II was coated with Vaseline^®.^ (Hindustan Unilever Ltd., Tamil Nadu, India). Group III was coated with GC Fuji VARNISH™ (GC Corporation, Tokyo, Japan). Group IV was coated with G-Coat Plus™ (GC Corporation, Tokyo, Japan). Group V was coated with EQUIA^®^ Coat (GC Corporation, Tokyo, Japan). The different surface protective agents used to coat the specimens are shown in Table 1. The detailed composition of different agents is shown in Table 2.

**Table 1.** Investigated surface protective agents, manufacturer, material type and lot numbers.

Material	Manufacturer	Material type
GC Fuji IX GP^®^ EXTRA	GC Corporation, Tokyo, Japan Lot number-1705161	Radiopaque Glass Ionomer restorative cement
GC Fuji VARNISH™	GC Corporation, Tokyo, Japan Lot number-1706281	Protective Coating
G-Coat Plus™	GC Corporation, Tokyo, Japan Lot number-1601061	Nanofilled self-adhesive light cured protective coating
EQUIA^®^ Coat	GC Corporation, Tokyo, Japan Lot number-1703091	Self-adhesive light cured wear resistant coating
Vaseline^®^	Hindustan Unilever Ltd., Tamil Nadu, India Lot number-B1662	Petroleum jelly

**Table 2.** The composition of each material based on manufacturer’s infosheet.

Product	Composition	Percentage
GC Fuji IX GP^®^ EXTRA	Powder:	
	• Strontium fluoroaluminosilicate glass	95%
	• Polyacrylic acid	5%
	Liquid:	
	• Aqueous Polyacrylic acid	40%
Vaseline^®^	Petrolatum	
GC Fuji VARNISH™	Isopropyl acetate	50–70%
	Acetone	20–30%
G-Coat Plus™	Methylmethacylate	50%
	Multifuctional methacrylate	
	Camphoroquinone	0.09%
EQUIA^®^ Coat	Methylmethacrylate	25–50%
	Photoinitiator	1–5%
	Synergist	1–5%
	Phosphoric acid ester monomer	1–5%

In Group II, Vaseline^®^ was applied with an applicator tip. In Group III, GC Fuji VARNISH™ was applied with the help of an applicator tip and then dried gently by blowing air with a chip blower. In Group IV and V the coating agents were applied with an applicator tip and were light cured with a visible light curing unit Bluephase^®^ N (Ivoclar Vivadent Marketing Pvt. Ltd., Mumbai, India) with a power output of 1200 mW/cm^2^ for 20 s. The different agents were placed in accordance with the manufacturers’ instructions.

### 2.1. Preparation of standard solutions

To prepare standard solutions two stock solutions were prepared: Stock solution A containing 200 µg/ml of methylene blue in nitric acid and stock solution B containing 20 µg/ml of methylene blue in nitric acid. In order to prepare stock solution A, 0.1 g of methylene blue was added in 500 ml of 65% nitric acid whereas to prepare stock solution B, 45 ml of 65% nitric acid was added to 5 ml of stock solution A. Finally, to prepare standard solutions, sequential amount of acid was added to stock solution B as shown in Appendix A (Supplementary material).

**Figure 1.**
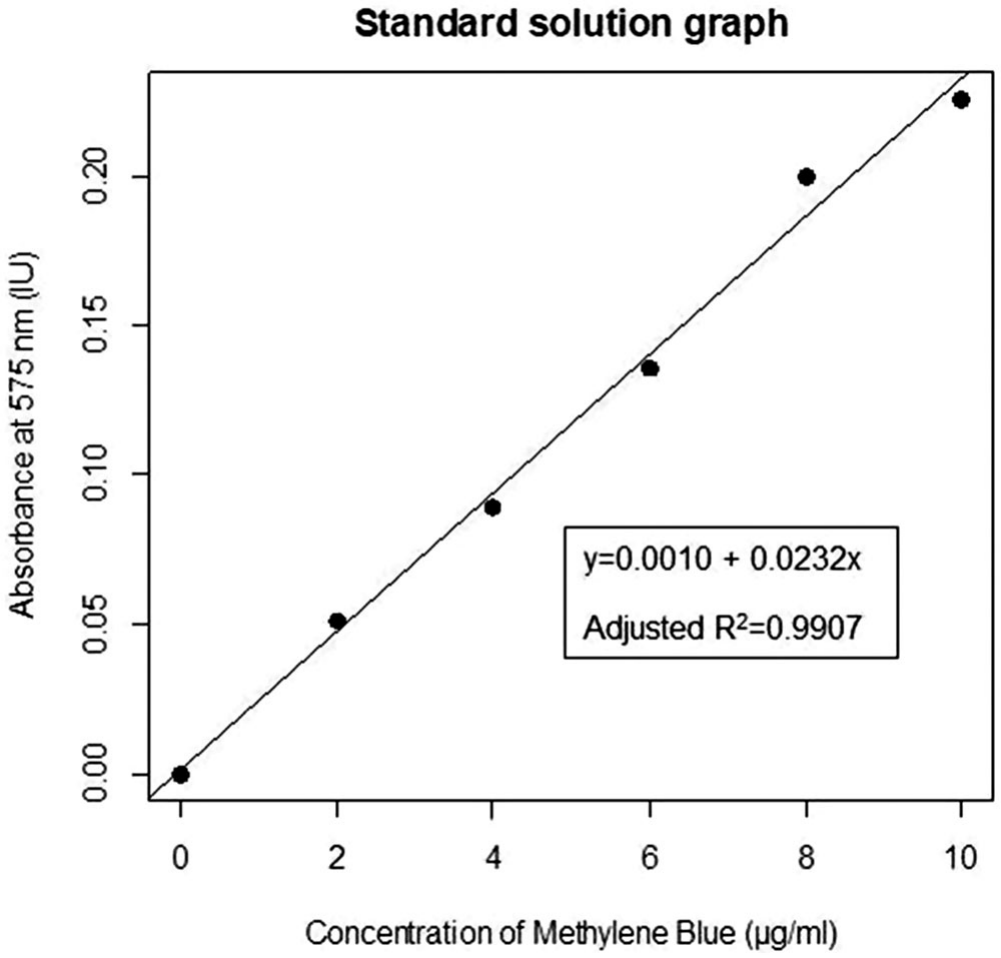
The linear relation between the different concentrations of methylene blue standard solutions and their respective absorbance values.

In order to obtain a linear regression equation and graph (Figure 1) one specimen was inserted into each of the six standard solutions and was allowed to dissolve for 36 h. The solutions were diluted with 2 ml of deionised water. After this, the solutions were filtered and centrifuged. The supernatant was used to determine the absorbance values using the Nanodrop 2000c Spectrophotometer (Thermo Fisher Scientific, Massachusetts, USA). A linear regression equation and graph (Figure 1) was determined using these values by R software.

### 2.2. Determination of effectiveness of surface protecton

The method used to quantify the effectiveness of surface protection was adapted from Serra et al. [13]. Following the surface treatments; each specimen was immersed in 0.05% methylene blue (Merck, Germany, CAS Number 7220-79-3) solution. After 24 h specimens were rinsed with 50 ml of deionised water (Grandlay Industries, Punjab, India). The coating was removed with a scalpel and the specimens were removed from the moulds. Following this, they were immersed separately into new tubes containing 1 ml of 65% nitric acid (Merck, Germany, CAS Number 7697-37-2). These solutions were referred to as experimental solutions. Specimens were completely dissolved after 36 h. The experimental solutions were diluted with 2 ml of deionised water. The solutions were filtered, centrifuged and the supernatant was used to determine the absorbance using the Nanodrop 2000c Spectrophotometer. The absorbance of standard and experimental solutions was scanned at wavelengths ranging from 500–800 nm and the best results were determined at 575 nm. The wavelength scans of the experimental solutions are provided in Appendix B (Supplementary material). The linear regression equation obtained from standard solutions was used to calculate the dye concentrations of the unknown experimental solutions. Data was analyzed using One-Way Analysis of Variance (ANOVA) [14] and Tukey’s Honest Significance Difference (Tukey’s HSD) [15] analysis packages in R software.

## 3. Results

**Figure 2.**
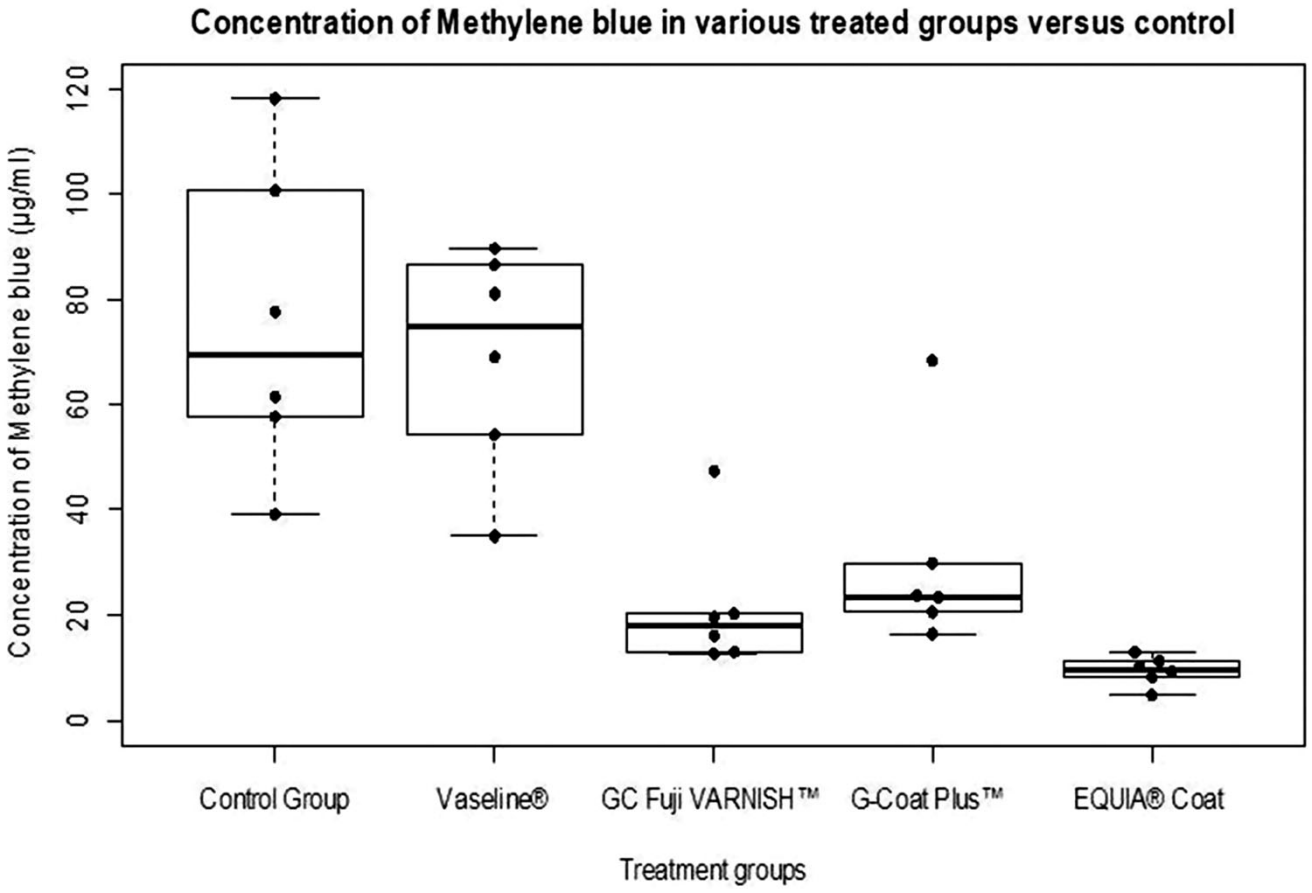
The concentrations of methylene blue in control versus treatment groups are displayed using the Box and Whisker plot overlaid with Beeswarm plot.

The concentration values of the experimental groups and the descriptive statistics of each experimental group are shown in the Table 3. The concentrations of methylene blue in different experimental groups were plotted (Figure 2) using R software.

**Table 3.** The descriptive statistics of each experimental group.

Group	Minimum	1^ST^ Quartile	Median	Mean	3^RD^ Quartile	Maximum
Control	39.05	58.72	69.53	75.81	94.92	118.28
Vaseline^®^	34.66	57.91	74.92	69.15	85.07	89.74
GC Fuji VARNISH™	12.41	13.49	17.59	21.25	19.84	47.20
G-Coat Plus™	16.51	21.16	23.54	30.39	28.23	68.62
EQUIA^®^ Coat	4.61	8.47	9.72	9.32	10.74	12.67

Note: unit for all values = µg/ml.

Replicate no. 4 from GC Fuji VARNISH™ and no. 2 from G-Coat Plus™ were found to be outliers. An outlier is defined as a data point that is located outside the whiskers of the boxplot (e.g. outside 1.5 times the interquartile range above the upper quartile and/or below the lower quartile). This finding of outliers was probably due to some experimental error.

The *p* value of less than 0.05 was considered statistically significant. ANOVA was highly significant (*p* = 3.71e−06). The intergroup comparison of the different groups was determined with Tukey’s HSD Test (Table 4). It shows that the difference between control and Vaseline was not statistically significant whereas there was a statistically significant difference when control was compared with G-Coat Plus™, GC Fuji VARNISH™ and EQUIA^®^ Coat. The three agents were equally effective in surface protection.

**Table 4.** Summary of Tukey HSD test.

Inter- group comparison	*p* Adjusted
Control- Vaseline^®^	.9739 N.S.
Control- GC Fuji VARNISH™	.0004***
Control- G-Coat Plus™	.0033**
Control- EQUIA^®^ Coat	.00002***
Vaseline^®^- GC Fuji VARNISH™	.00199**
Vaseline^®^- G-Coat Plus™	.0143*
Vaseline^®^- EQUIA^®^ Coat	.0001***
GC Fuji VARNISH™- G-Coat Plus™	.9265 N.S.
GC Fuji VARNISH™- EQUIA^®^ Coat	.8168 N.S.
G-Coat Plus™- EQUIA^®^ Coat	.3527 N.S.

N.S.: not significant. *p ≤ .05; **p ≤ .01; ***p ≤ .001.

## 4. Discussion

In this study, the microleakage of dye was measured with a spectrophotometer to compare different agents. The method was first reported by Douglas and Zakariasen (1981) [16]. The advantage of this method is that it is a quantitative method and thus eliminates errors in subjective operator evaluations used in qualitative measurements [17]. This method utilises the Beer Law to measure the concentration of dye by measuring the wavelength of light [18].

In this study the concentration of dye penetration into the specimens was evaluated after 24 h of storage in the dye solution as the cross linking process continues for one day after mixing [19]. The results of the study showed that the Vaseline^®^ group was comparable to controls. This may be due to ease of washing away of Vaseline^®^ [20]. This is in accordance with the results of previous studies done by Booth et al. [21].

The GC Fuji VARNISH™ was effective as a surface protectant. This finding was supported by a study done by Nicholson et al. where they concluded that the application of varnish led to reduction in water loss irrespective of the fact whether the varnish applied was an unsophisticated lacquer or a more chemically advanced light curable formulation [22].

The better performance of G-Coat Plus™ as compared to Vaseline^®^ can be attributed to its property of sealing the micro-gaps with nanosized filler particles [23]. The results suggest that the EQUIA^®^ Coat was a very effective surface protective agent. This is in accordance with the results obtained by Klinke et al. in which they concluded that the overall superior performance of EQUIA^®^ Coat can be attributed to the nanofilled surface coating agent which led to primary stabilization of the restorative material and fills all the superficial surface defects [24]. According to Bagheri et al., the advantage of self-adhesive coating agents is that it provides a lamination effect on GIC surface and facilitates complete maturation of GIC by preventing early contact with extrinsic water, and therefore creates a stronger material [25]. It forms a thin layer of coating agent and is wear resistant [26]. As claimed by the manufacturer, the performance of EQUIA^®^ Coat can be attributed to its new crosslinking monomer chemistry, which led to improved polymerization and created a tougher resin matrix reinforced by mono dispersion nano filler technology. EQUIA^®^ Coat was more flowable than G-Coat Plus™, which led to a smoother surface. The coating has an additional advantage that it acts like a glaze and further enhanced the aesthetics of the restorative material. The other properties of EQUIA^®^ Coat, which explained its clinical performance were that it was highly hydrophilic and possessed extremely low viscosity which led to superior surface seal [1].

The dye penetration behaviour of light cured resins can be attributed to the cure process because the dental cure lamp used has a power output of 1200 mW/cm^2^. The lamp can generate a reasonable amount of heat, which is expected to accelerate the setting reaction in the surface layers of the specimens, and contribute to strength and structure [12]. It has been shown that thermo light curing improves the micro hardness, reduces the microleakage and improves the success outcome of the GIC restoration [27].

To ensure that surface smoothness did not affect the results, all specimens were prepared using mylar strips as it was suggested that the smoothest surfaces of GIC were produced with the use of mylar strips [28].

Initial setting occurs within three to four minutes, but precipitation, gelation and hydration continues for at least 24 h and setting continues slowly for much longer periods [29]. But the present study has the limitation of recording the effect of surface protectant for only 24 h. The study also has the limitation of not recognizing the effect of different finishing agents on the dye penetration and the effect of surface protective agents on fluoride release.

## 5. Conclusion

In the present study, the materials demonstrated the following order of increasing efficiency: Control = Vaseline^®^ < G-Coat Plus™ = GC Fuji VARNISH™ = EQUIA^®^ Coat. There is no significant difference between GC Fuji VARNISH™, G-Coat Plus™ and EQUIA^®^ Coat. As far as cost was concerned, during the study it was observed that GC Fuji VARNISH™ was the most cost-effective agent compared to G Coat Plus™ and EQUIA^®^ Coat, which were the expensive options with similar performance.

## Supplementary Material

Supplemental Material
