# Supplementary material for: A comparative evaluation of resin- and varnish-based surface protective agents on glass ionomer cement – a spectrophotometric analysis
Source: Biomater Investig Dent. 2020 Jan 11;7(1):25–30. doi: 10.1080/26415275.2020.1711760 (PMC7006751; doi:10.1080/26415275.2020.1711760)

**SUPPLEMENTARY MATERIAL**

**Appendix A**

**Composition of standard solutions**

| **TITLE** | **CONCENTRATION** | **COMPOSITION** |
| --- | --- | --- |
| Standard solution S0 | 0µg/ml | 10 ml acid + 0 ml stock solution B |
| Standard solution S2 | 2µg/ml | 9 ml acid + 1 ml stock solution B |
| Standard solution S4 | 4µg/ml | 8 ml acid + 2 ml stock solution B |
| Standard solution S6 | 6µg/ml | 7 ml acid + 3 ml stock solution B |
| Standard solution S8 | 8µg/ml | 6 ml acid + 4 ml stock solution B |
| Standard solution S10 | 10µg/ml | 5 ml acid + 5 ml stock solution B |

**Appendix B**

**Wavelength scans for the Vaseline^®^ coated samples (6 replicates).**


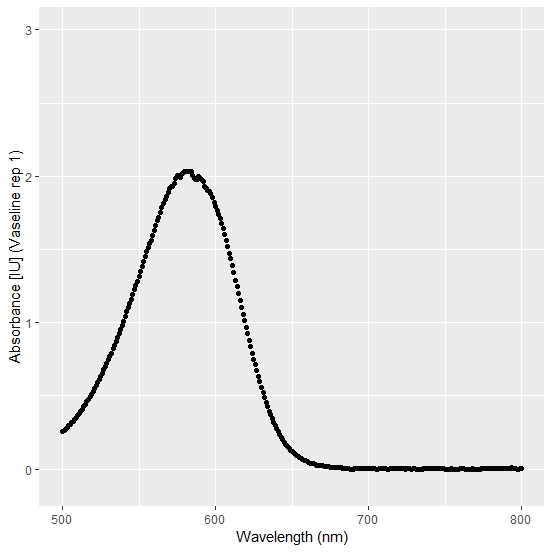

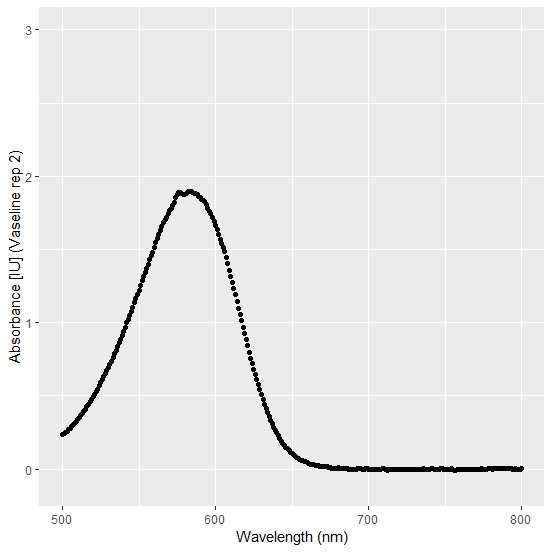

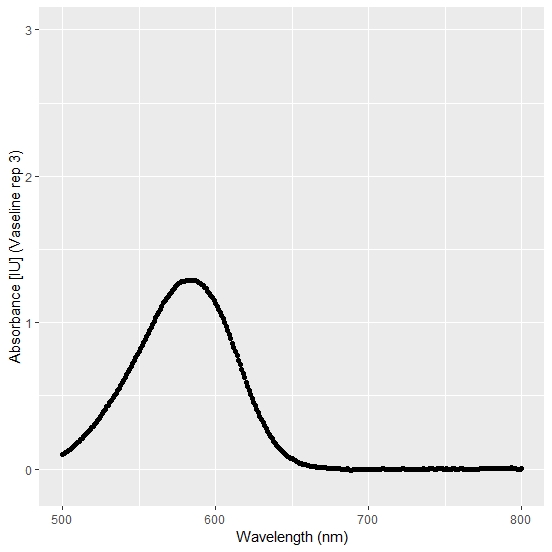

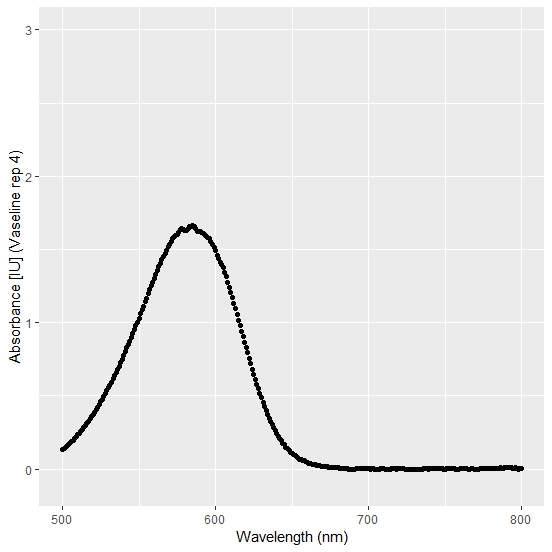

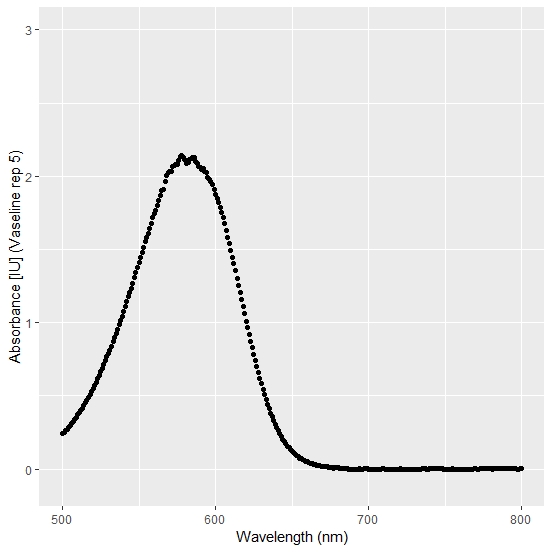

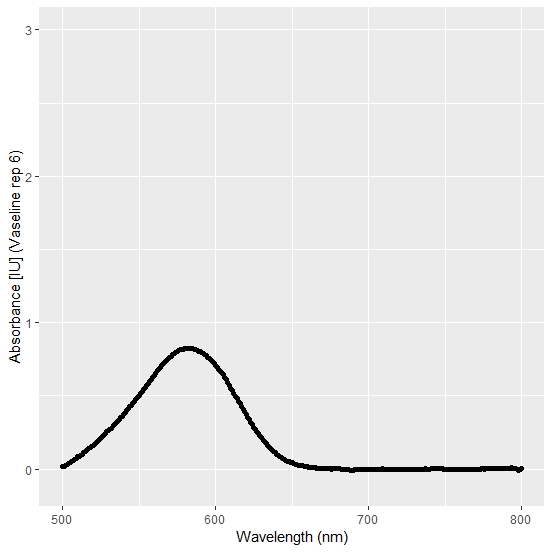


**Wavelength scans for the GC Fuji VARNISH™ coated samples (6 replicates).**


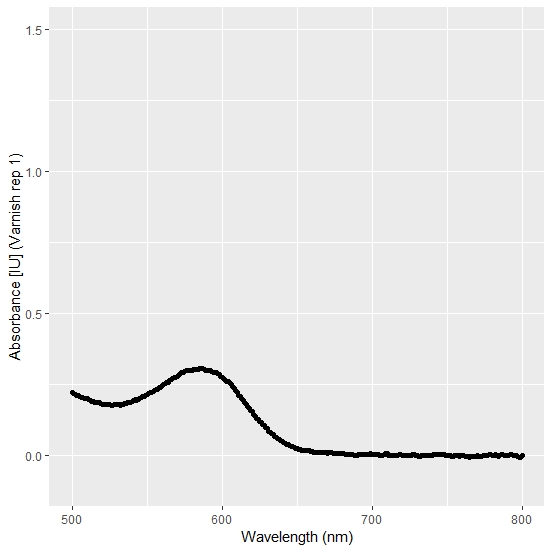

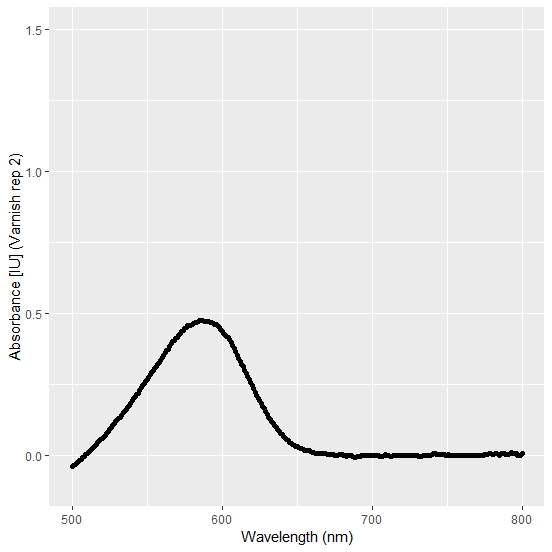

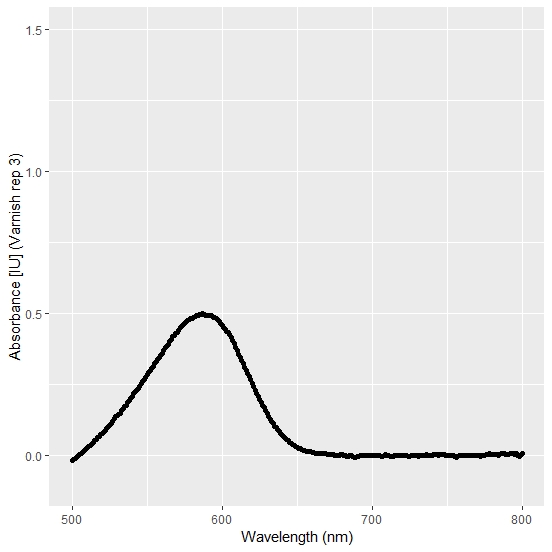

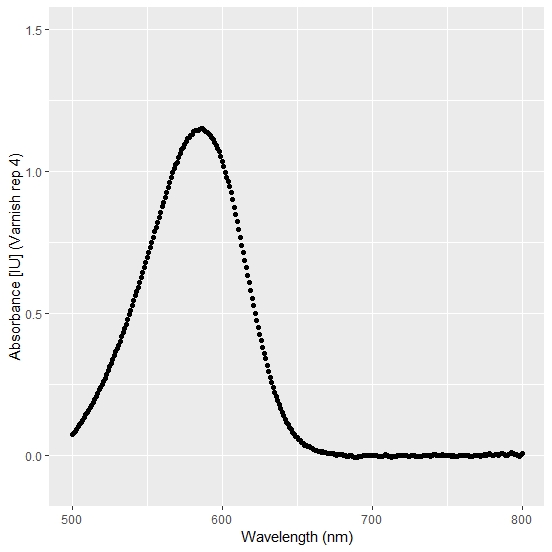

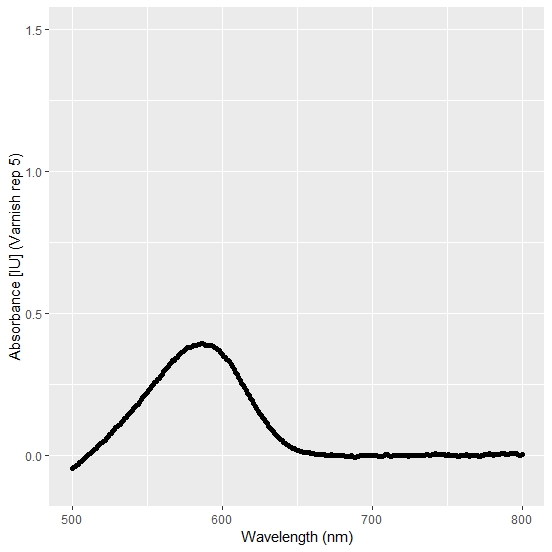

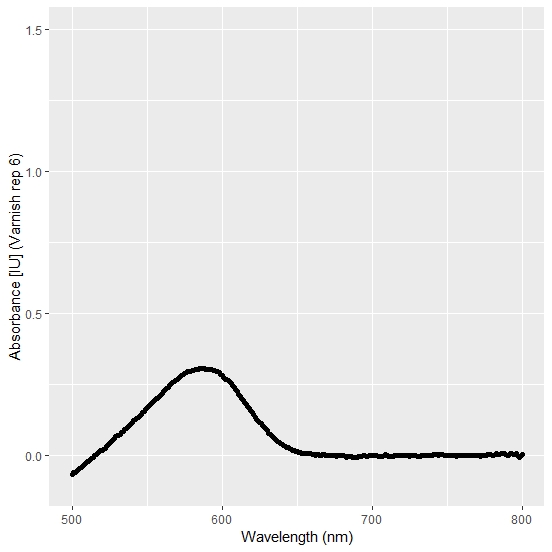


**Wavelength scans for the G-Coat Plus™ coated samples (6 replicates).**


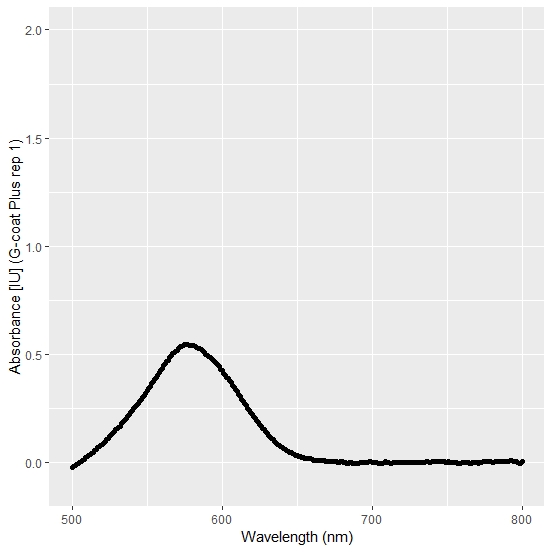

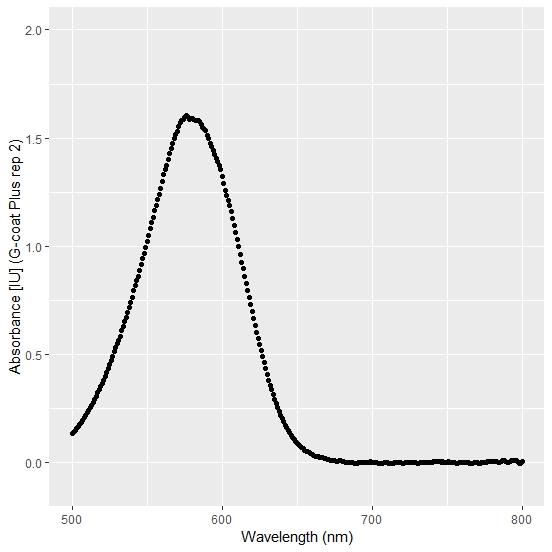

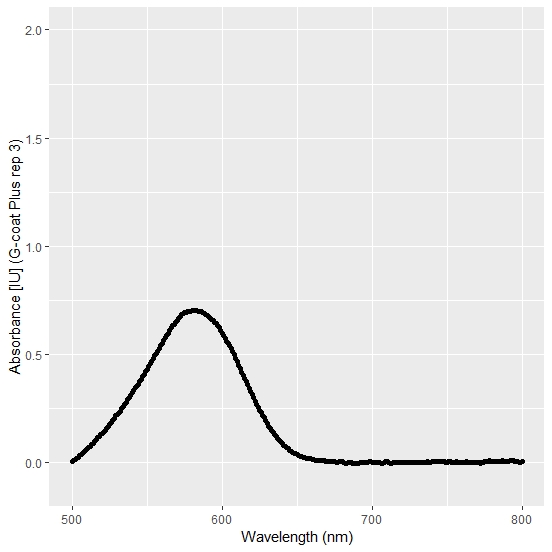

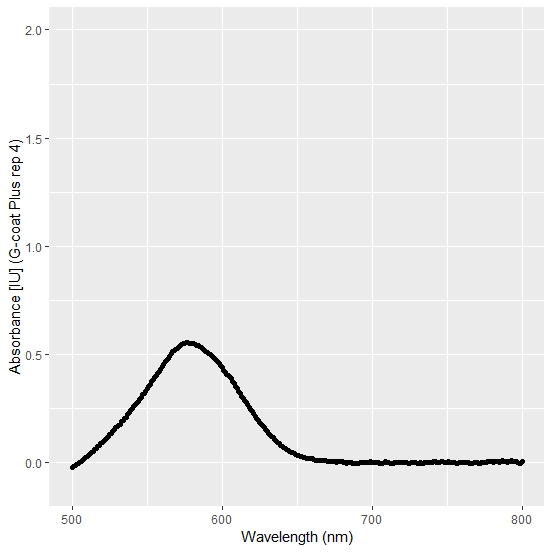

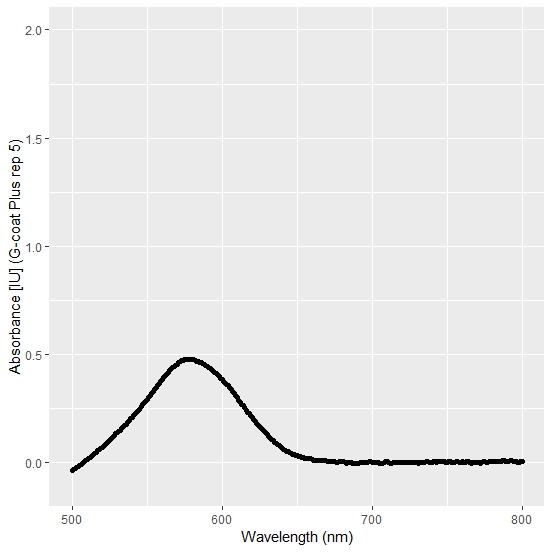

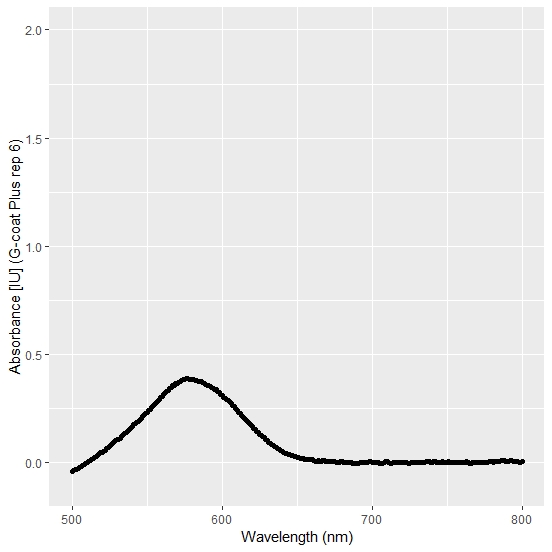


**Wavelength scans for the EQUIA^®^ Coat coated samples (6 replicates).**
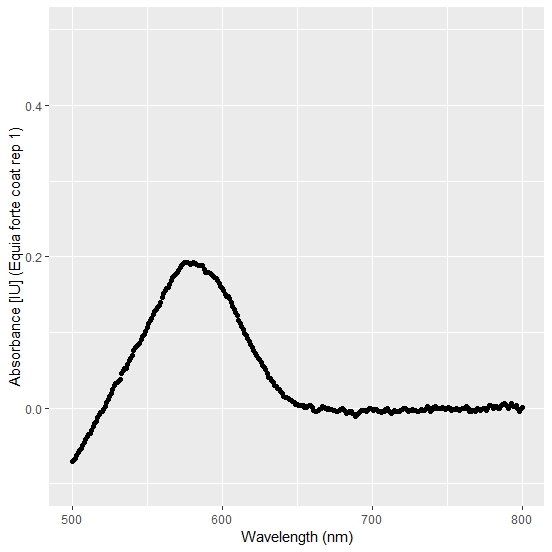

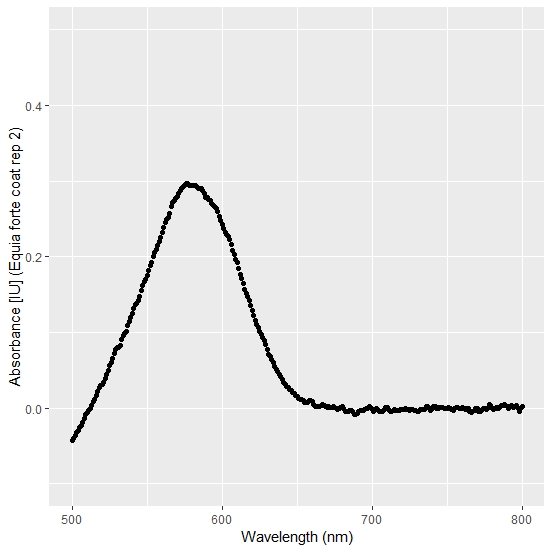

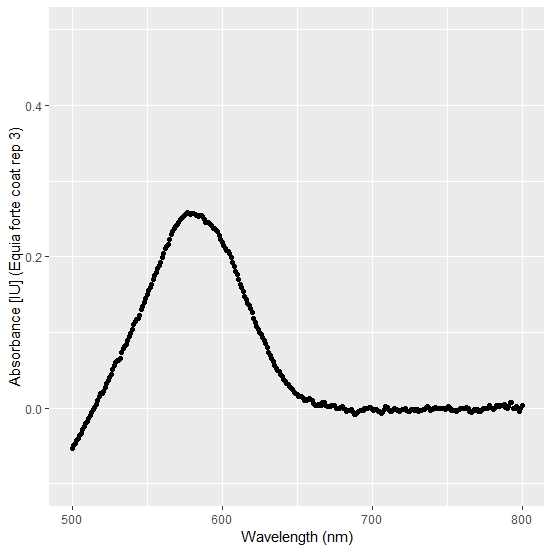

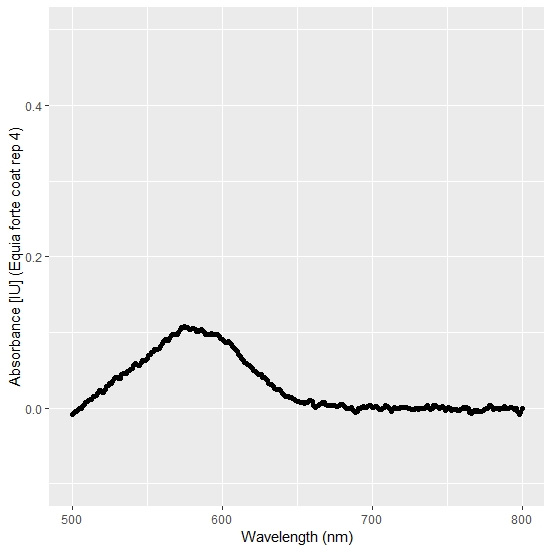

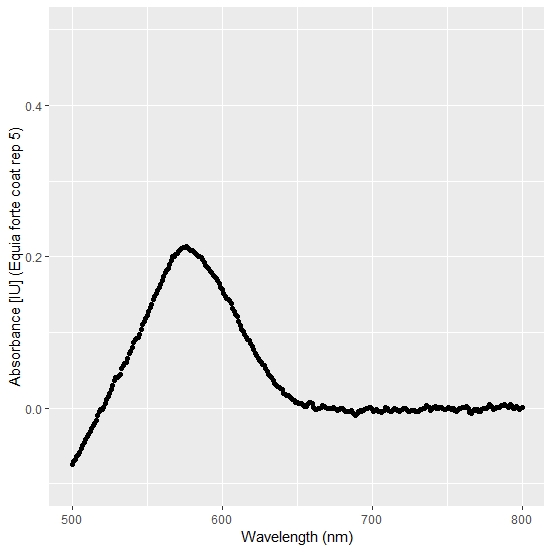

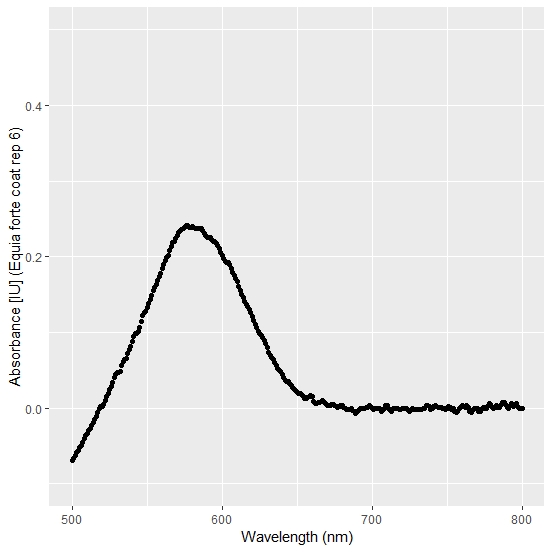

Supplement: Supplemental Material [file IABO_A_1711760_SM1760.docx]
